# Amelioration of prevalence of threatened preterm labor during the COVID-19 pandemic: nationwide database analysis in Japan

**DOI:** 10.1038/s41598-022-19423-x

**Published:** 2022-09-12

**Authors:** Mizuki Ohashi, Shunichiro Tsuji, Sachiko Tanaka-Mizuno, Kyoko Kasahara, Makiko Kasahara, Katsuyuki Miura, Takashi Murakami

**Affiliations:** 1 Department of Obstetrics and Gynecology, Shiga University of Medical Science, Seta Tsukinowa-Cho, Otsu, Shiga 520-2192 Japan; 2 NCD Epidemiology Research Center, Shiga University of Medical Science, Otsu, Japan; 3 Department of Digital Health and Epidemiology, Graduate School of Medicine and Public Health, Kyoto University, Kyoto, Japan; 4 Department of Public Health, Shiga University of Medical Science, Otsu, Japan

**Keywords:** Paediatric research, Viral infection

## Abstract

We aimed to evaluate the changes in maternal and neonatal complications such as threatened preterm labor (TPL) and preterm birth before and during the coronavirus disease 2019 (COVID-19) pandemic using large-scale real-world data in Japan. We obtained data from the Japan Medical Data Center claims database and evaluated differences in maternal and neonatal complications, such as the prevalence of TPL and preterm birth before the COVID-19 pandemic (in the year 2018 or 2019) and during the COVID-19 pandemic (in 2020). We included 5533, 6257, and 5956 deliveries in the years 2018, 2019, and 2020, respectively. TPL prevalence and preterm birth had significantly decreased in 2020 (41.3%, 2.6%, respectively) compared with those reported in 2018 (45.3%, 3.9%, respectively) and 2019 (44.5%, 3.8%, respectively). Neonatal outcomes such as low-birth-weight infants and retinopathy of prematurity were also improved during the pandemic. There were no clear trends in the prevalence of maternal complications such as hypertensive disorders of pregnancy; hemolysis, elevated liver enzymes, and low platelets (HELLP) syndrome; and preeclampsia. Oral ritodrine hydrochloride usage in all participants had significantly decreased during the COVID-19 pandemic. In conclusion, our results suggest that the COVID-19 pandemic has ameliorated TPL and consequently reduced the number of preterm births.

## Introduction

The coronavirus disease 2019 (COVID-19) has become a great public health concern. After the World Health Organization declared the COVID-19 outbreak a pandemic, some studies reported that people’s lifestyles have become more sedentary and less active worldwide^1^ and also in Japan^2^. People tend to stay at home and avoid outdoor and excessive leisure-time activities. The resulting inactivity was observed across age groups and even among pregnant women. Such lifestyle changes due to the pandemic may affect the risk of maternal and neonatal complications, such as threatened preterm labor (TPL) and preterm birth.

The association between maternal physical activity and TPL is a controversial topic of great scientific interest. Increased participation in leisure-time physical activities was previously linked to a lower risk of preterm birth^3^. In contrast, participation in heavy, long, or standing occupational physical activities was also shown to be associated with an increased risk of preterm birth^4–7^. The influence of physical activity on TPL and preterm birth are different according to the types and intensity of activities. Bed rest is one of the most common interventions to prevent preterm birth. However, its effectiveness for TPL prevention remains unclear^8–10^. Besides heavy labor during pregnancy, previous history of TPL or preterm birth^11–13^ and infections^7,11,14^ are also known risk factors of TPL and preterm birth.

Although the use of ritodrine hydrochloride is generally avoided for TPL treatment in other countries due to cardiopulmonary side effects, its use is common in Japan^9,10,15^. However, there is no report of the impact of the use of ritodrine hydrochloride during the COVID-19 pandemic. Because TPL management may also have been influenced by the COVID-19 pandemic through maternal lifestyle changes, the correlation between the effect of COVID-19 and the use of tocolytic agents is unclear. However, it is difficult to evaluate TPL and its management in a nationwide context in Japan during the COVID-19 pandemic with a limited number of patients in a few hospitals. Therefore, we aimed to evaluate the changes in the occurrence of maternal and neonatal complications, such as TPL, in response to lifestyle changes due to the COVID-19 pandemic by assessing large-scale real-world data in Japan.

## Methods

### Data source and study participants

We extracted information from the Japan Medical Data Center database (JMDC, Inc., Tokyo, Japan; JMDC). This database includes the patient's health insurance claims, medical examination data, and ledger data from employee-based insurances in Japan.

We included women who delivered their babies between October and December in 2018, 2019, and 2020 in this study. As the Japanese government declared a state of emergency in April 2020 for the first time since the global COVID-19 outbreak, we defined the deliveries in 2018 and 2019 as those that occurred before the COVID-19 pandemic and the deliveries in 2020 as those that occurred during the pandemic. We limited the deliveries from October to December to set the entire pregnancy period after April 2020, when people started to adopt a sedentary lifestyle. Deliveries were defined using the year and month of birth of the participant’s newborn based on the ledger data or procedure codes and dates related to deliveries. From the JMDC database, we extracted 17,746 deliveries, which met our inclusion criteria. This study was conducted following proper guidelines, such as the Japanese Ethical Guidelines for Medical and Biological Research Involving Human Subjects. Informed consent from participants was not required as all identifying information of the participants was completely anonymized. The study protocol was approved by the Ethics Committee at the Shiga University of Medical Science (No. R2021-139).

### Definition of variables

TPL and other complications were diagnosed based on the International Classification of Diseases, Tenth Revision (ICD-10) codes. Procedures, such as cesarean section, were searched by Japanese standardized procedure codes. Data on the use of medication, such as ritodrine hydrochloride and magnesium sulfate, were retrieved using Japanese individual drug codes. The amount of medication used was calculated based on the prescribed dosage for each medicine and dosing period.

TPL was defined by the ICD-10 code for TPL (O60.0) or as the usage of any type of ritodrine hydrochloride. Preterm birth was defined by the newborn’s ICD-10 code for preterm birth (P07.2, P07.3) and the birth of a very low or extremely low birth weight infant (P07.0: birth weight of less than 1000 g, P07.1: 1000–1499 g).

Complications diagnosed during pregnancy—such as hypertensive disorders of pregnancy (O10, O11, O13–O16); hemolysis, elevated liver enzymes, and low platelets (HELLP) syndrome (O14.2); and preeclampsia (O14.0, O14.1, O14.9)—were extracted using ICD-10 codes recorded for diagnoses within seven months before delivery. Data on complications diagnosed before pregnancy, such as hypertension (I10-I15), diabetes (E10-E14), and anti-phospholipid syndrome (D68.6), were extracted using ICD-10 codes diagnosed more than ten months before delivery. Data on neonatal outcomes were extracted using ICD-10 codes for respiratory distress syndrome (P22.0), low birth weight (P07.0-P07.2) and retinopathy of prematurity (H35.1), procedure codes for admission to neonatal intensive care unit and respiratory support, or drug codes for artificial pulmonary surfactant, which were recorded in the delivery month.

### Statistical analysis

We evaluated differences in maternal and neonatal complications at childbirth between October and December in 2018, 2019, and 2020. Comparison analysis was performed using Pearson’s chi-square test for categorical variables and Student’s t-test or Wilcoxon’s rank sum test for continuous variables. Bonferroni-corrected p-value of less than 0.0167 was used as the statistical significance threshold due to multiple testings in 2018, 2019, and 2020. All statistical analyses were performed using SAS version 9.42 (SAS Institute Inc., Cary, NC, USA).

## Results

**Figure 1:**
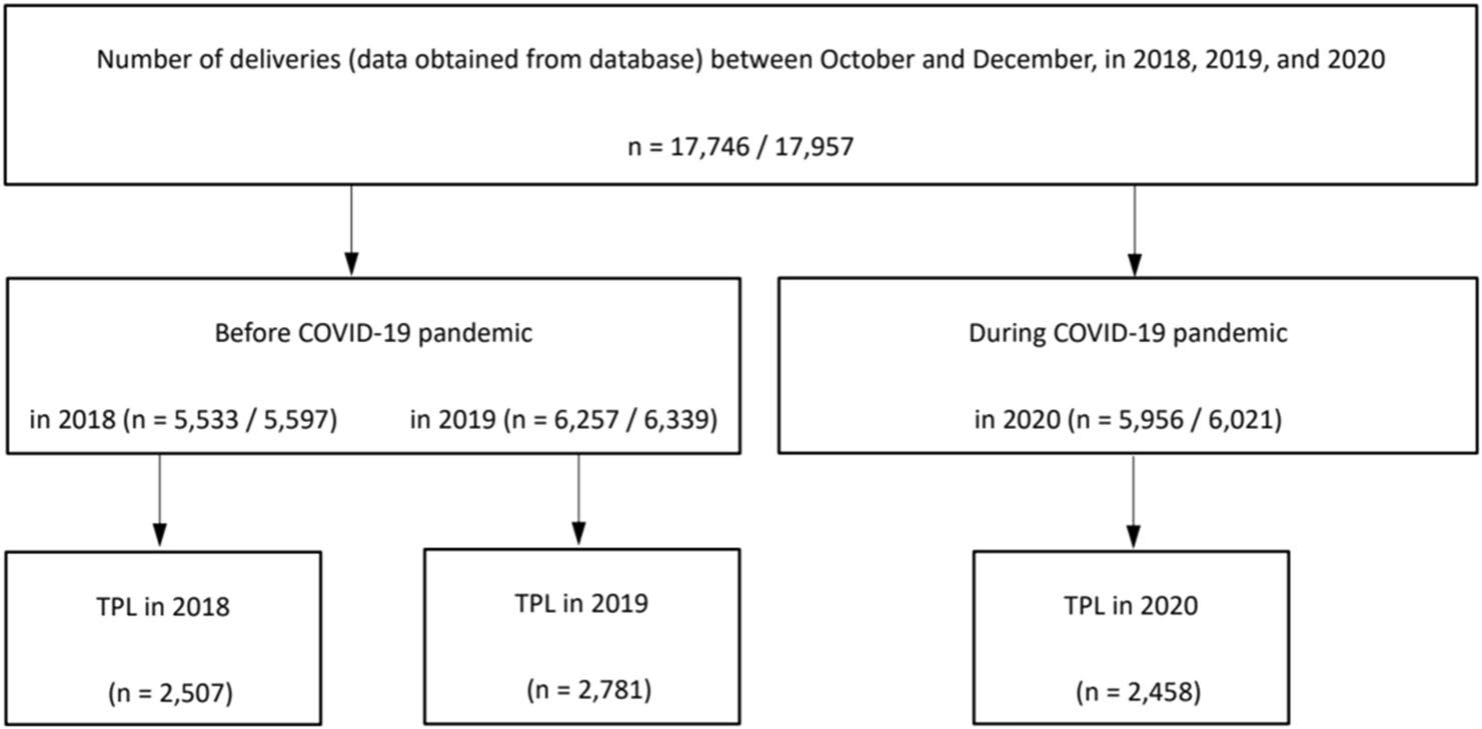
Flow chart of the study population. We firstly extracted delivery information from October to December between 2018 and 2020 from the database. The numbers of participants were shown based on deliveries and newborns. We defined the deliveries in 2018 and 2019 as before the COVID-19 pandemic and the deliveries in 2020 as during the COVID-19 pandemic. TPL was diagnosed by ICD-10 code and the use of tocolytic agents, which records were used to analyze each medical use and dosage. *COVID-19* Coronavirus disease 2019, *TPL* threatened preterm labor.

There were 5533, 6257, and 5956 deliveries in the years 2018, 2019, and 2020, respectively (Fig. 1); the corresponding number of newborns were 5597, 6339, and 6021, respectively (Fig. 1). Basic characteristics of participants, such as mother’s age at birth or complications before pregnancy, including hypertension, had not changed between periods before and during the COVID-19 pandemic (Table 1). TPL prevalence had significantly decreased in 2020 (n = 2458, 41.3%) compared with that in 2018 (n = 2507, 45.3%) and 2019 (n = 2781, 44.5%) (Table 1). Preterm birth prevalence in 2020 (n = 154, 2.6%) had declined in comparison to those in 2018 (n = 218, 3.9%) and 2019 (n = 238, 3.8%). Meanwhile, no clear trends were observed in the prevalence of hypertensive disorders of pregnancy, HELLP syndrome, preeclampsia, emergency transfer, and deliveries by cesarean section. Among the participants during the COVID-19 pandemic, we extracted seven COVID-19 cases by ICD-10 code (U07.1), which were four cases in the TPL group and three cases in the non-TPL group (data not shown). No participants got vaccinations in our study as the COVID-19 vaccinations had not been approved yet in Japan in 2020.

**Table 1:** Maternal characteristics and complications during pregnancy among the 17,746 deliveries in the years 2018, 2019, and 2020.

	Year of childbirth						
	Before COVID-19 pandemic				During COVID-19 pandemic		
	2018	2019		p-value^a^	2020	p-value^b^	p-value^c^
Number of deliveries (n)	5533	6257			5956		
Childbirths of singleton (n, %)	5469 (98.8)	6177 (98.7)		0.548	5892 (98.9)	0.675	0.296
Mother's age at childbirth	32.6 (4.6)	32.8 (4.6)		0.112	32.8 (4.6)	0.038	0.601
**Complications diagnosed before pregnancy**							
Hypertension (n, %)	97 (1.8)	110 (1.8)		0.984	108 (1.8)	0.808	0.818
Diabetes (n, %)	191 (3.5)	212 (3.4)		0.849	204 (3.4)	0.937	0.911
Anti-phospholipid antibody syndrome (n, %)	31 (0.6)	54 (0.9)		0.053	39 (0.7)	0.515	0.186
Threatened preterm labor (n, %)	2507 (45.3)	2781 (44.5)		0.347	2458 (41.3)	< 0.001	< 0.001
Use of ritodrine (n, %)	1797 (32.5)	2014 (32.2)		0.737	1722 (28.9)	< 0.001	< 0.001
Use of magnesium sulfate (n, %)	107 (1.9)	128 (2.1)		0.665	111 (1.9)	0.783	0.468
Preterm birth (n, %)	218 (3.9)	238 (3.8)		0.690	154 (2.6)	< 0.001	< 0.001
Hypertensive disorders of pregnancy (n, %)	302 (5.5)	414 (6.6)		0.009	410 (6.9)	0.002	0.556
HELLP syndrome (n, %)	7 (0.13)	9 (0.14)		0.799	2 (0.03)	0.098	0.042
Preeclampsia (n, %)	249 (4.5)	341 (5.5)		0.018	329 (5.5)	0.012	0.858
Emergency transfer (n, %)	128 (2.3)	154 (2.5)		0.600	146 (2.5)	0.628	0.972
Cesarean section (n, %)	1206 (21.8)	1440 (23.0)		0.114	1284 (21.6)	0.757	0.053

The denominator for the percentage is the number of deliveries. Mother’s age at childbirth is presented as mean (standard deviation).

P-values of Pearson’s chi-square test or Student’s t-test a: between 2018 and 2019, b: between 2018 and 2020, and c: between 2019 and 2020 are shown. We used a P value of 0.0167 to indicate significance to correct for multiplicity.

In terms of neonatal complications, the prevalence of low-birth-weight infant had significantly decreased in 2020 (n = 224, 3.7%) compared with those in 2018 (n = 330, 5.9%) and 2019 (n = 353, 5.6%) (Table 2). The prevalence of retinopathy of prematurity had also decreased in 2020 (n = 14, 0.2%) compared with those in 2018 (n = 37, 0.7%) and 2019 (n = 41, 0.7%). There were no differences in admission to the neonatal intensive care unit and diagnosis of infant respiratory distress syndrome between the periods before and during the COVID-19 pandemic.

**Table 2:** Neonatal characteristics and complications among the 17,957 newborns that were born in the years 2018, 2019, and 2020.

	Year of childbirth					
	Before COVID-19 pandemic			During COVID-19 pandemic		
	2018	2019	p-value^a^	2020	p-value^b^	p-value^c^
Number of newborns (n)	5597	6339		6021		
Preterm birth (n, %)	218 (3.9)	238 (3.8)	0.690	154 (2.6)	< 0.001	< 0.001
Admission to neonatal intensive care unit (n, %)	190 (3.4)	251 (4.0)	0.103	168 (2.8)	0.060	< 0.001
Respiratory distress syndrome (n, %)	413 (7.4)	549 (8.7)	0.010	395 (6.6)	0.083	< 0.001
Respiratory support (n, %)	178 (3.2)	242 (3.8)	0.059	159 (2.6)	0.083	< 0.001
Use of artificial pulmonary surfactant (n, %)	39 (0.7)	58 (0.9)	0.185	35 (0.6)	0.434	0.032
Low birth weight (n, %)	330 (5.9)	353 (5.6)	0.442	224 (3.7)	< 0.001	< 0.001
Retinopathy of prematurity (n, %)	37 (0.7)	41 (0.7)	0.923	14 (0.2)	< 0.001	< 0.001

The denominator for the percentage is the number of newborns.

P-values of Pearson’s chi-square test, a: between 2018 and 2019, b: between 2018 and 2020, and c: between 2019 and 2020 are shown. We used a P value of 0.0167 to indicate significance to correct for multiplicity.

Oral ritodrine hydrochloride usage in total participants was significantly decreased in 2020 (n = 1626, 27.3%) compared to those in 2018 (n = 1720, 31.1%) and 2019 (n = 1911, 30.5%). For intravenous administration of ritodrine hydrochloride, there was no significant difference among the years of 2018 (n = 364, 6.6%), 2019 (n = 424, 6.8%), and 2020 (n = 396, 6.7%). In patients with TPL, the ritodrine hydrochloride usage rate, for both intravenous and oral administration, did not significantly change in the years 2018 (14.5% for intravenous, 68.6% for oral administration), 2019 (15.3%, 68.7%), and 2020 (16.1%, 66.2%). Usage rate of magnesium sulfate also did not show any differences among the years 2018 (3.4%), 2019 (3.0%), and 2020 (3.2%) in patients with TPL. The median intravenous dosage of ritodrine hydrochloride (50 mg of ritodrine hydrochloride per ampule) did not change in 2020 (22.0 ampoules per delivery) compared with those in 2018 (21.5 ampoules per delivery) and 2019 (20.0 ampoules per delivery) (Table 3). The median oral dosage of ritodrine hydrochloride (5 mg of ritodrine hydrochloride per tablet) also did not change in 2020 (62.5 tablets per delivery) compared with those in 2018 (63.0 tablets per delivery) and 2019 (63.0 tablets per delivery). The dosage of magnesium sulfate (10 g of magnesium sulfate per ampule) did not present any difference in 2020 (8.0 ampoules per delivery) compared with those in 2018 (8.0 ampoules per delivery) and 2019 (7.0 ampoules per delivery). The percentage of TPL treated in larger medical institutions (≥ 20 beds) did not change in the years 2018 (46.0%), 2019 (45.0%), and 2020 (43.7%).

**Table 3:** Use of tocolytic agents in 2018, 2019, and 2020 among all deliveries and cases with threatened preterm labor.

	Year of childbirth					
	Before COVID-19 pandemic			During COVID-19 pandemic		
	2018	2019	p-value^c^	2020	p-value^d^	p-value^e^
Number of deliveries (n)	5533	6257		5956		
Use of ritodrine for oral administration^a^ (n, %)	1720 (31.1)	1911 (30.5)	0.523	1626 (27.3)	< 0.001	< 0.001
Use of ritodrine for intravenous administration^a^ (n, %)	364 (6.6)	424 (6.8)	0.668	396 (6.7)	0.880	0.778
Number of threatened preterm labor cases (n)	2507	2781		2458		
Use of ritodrine hydrochloride						
For oral administration^b^ (n, %)	1720 (68.6)	1911 (68.7)	0.932	1626 (66.2)	0.065	0.048
Dosage for oral administration (tablets per delivery)	63.0 (28.0, 141.0)	63.0 (28.0, 133.0)	0.263	62.5 (21.0, 132.0)	0.064	0.430
For intravenous administration^b^ (n, %)	364 (14.5)	424 (15.3)	0.459	396 (16.1)	0.120	0.390
Dosage for intravenous administration (ampoules per delivery)	21.5 (6.5, 66.0)	20.0 (6.0, 58.0)	0.290	22.0 (6.0, 62.0)	0.663	0.573
Use of magnesium sulfate^b^ (n, %)	84 (3.4)	83 (3.0)	0.447	78 (3.2)	0.725	0.693
Dosage of magnesium sulfate (ampoules per delivery)	8.0 (4.0, 32.0)	7.0 (3.0, 31.0)	0.787	8.0 (4.0, 37.0)	0.507	0.283
Threatened preterm labor treated in larger medical institutions (≥ 20 beds)^b^ (n, %)	1153 (46.0)	1250 (45.0)	0.447	1075 (43.7)	0.110	0.378

Uses of medicines are described in number (a: % for the number of deliveries, b: % for the number of threatened preterm labor cases), and their dosages are described in median (25th percentile, 75th percentile).

P-values of Pearson’s chi-square test or Wilcoxon’s rank sum test c: between 2018 and 2019, d: between 2018 and 2020, and e: between 2019 and 2020 are shown. We used a P value of 0.0167 to indicate significance to correct for multiplicity.

## Discussion

The present study showed that the prevalence of TPL and preterm birth during the COVID-19 pandemic significantly decreased compared with those before the COVID-19 pandemic. Neonatal outcomes, such as low-birth-weight infants and retinopathy of prematurity, were also improved during the COVID-19 pandemic. Our result is consistent with a previous report of decreased preterm birth during the pandemic in high-income countries^16^. Although another study also reported that the prevalence of TPL and preterm birth decreased during and after the state of emergency in Japan, we also demonstrated improved outcomes of neonatal complications based on a larger sample size compared to the previous study^17^. Furthermore, our study showed oral ritodrine hydrochloride usage in all the participants significantly decreased during the COVID-19 pandemic.

After the first state of emergency declaration in April 2020 by the Japanese government, people changed their lifestyles to stay and work at home in Japan^2^. Increases in sedentary behaviors during lockdowns in several countries were also reported^1^. The association between physical activity and TPL is still unclear and varies according to the types and intensity of physical activity, such as leisure-time or occupational physical activity^3–7^. Additionally, the efficacy of bed rest in TPL management remains to be determined^8–10^. Our result showed that the lifestyle change during the COVID-19 pandemic might influence TPL prevention. However, it is difficult to determine whether there is a direct impact of reduced physical activity on TPL. The types of reduced physical activity due to the COVID-19 pandemic may vary depending on personal lifestyle or occupation. In Japan, while people avoided going outside and excess physical activity during the COVID-19 pandemic, necessary activities of daily living and working were not restricted. Therefore, it is difficult to assume how much people reduced physical activity in our study.

Maternal complications such as hypertensive disorders of pregnancy can be a cause of medically indicated preterm birth^11^. However, there were no significant differences in the prevalences of hypertensive disorders of pregnancy, HELLP syndrome, and preeclampsia between periods before and during the COVID-19 pandemic in our study. A previous systematic review indicated that there are discrepancies with regard to the influence of sedentary behavior on hypertensive disorders^18^. One study showed no significant difference in the prevalence of hypertensive disorders between periods before and during the COVID-19 pandemic^16^. Other studies mentioned that appropriate physical activity reduced the risk of gestational hypertensive disorders^19^ and preeclampsia^20^. It is possible that reduced physical activities during the COVID-19 pandemic are not drastic enough to increase the risk of hypertensive disorders. During the pandemic, heavy labor and excess leisure-time activities are limited while necessary daily activities are not reduced. This setting might represent a favorable environment for pregnant women to reduce TPL and not increase hypertensive disorders. Nevertheless, we need to carefully consider the influence of a sedentary lifestyle on hypertensive disorders of pregnancy and other complications during pregnancy.

We did not investigate maternal mental health problems such as depression. However, the COVID-19 pandemic may adversely impact maternal mental health by reduction of physical activity and restriction on social activities, such as meeting people or utilizing social support. A previous report showed no significant influence of a sedentary lifestyle on depression^18^. In contrast, other studies showed that maternal depression increased during the pandemic in several countries^16,21^. Maternal depression during pregnancy can be a risk of preterm birth^11^. Therefore, maternal mental healthcare under limited social support due to COVID-19 is another important public health concern.

We also revealed that only the oral usage rate of ritodrine hydrochloride in all participants significantly decreased during the COVID-19 pandemic. TPL management by the use of ritodrine hydrochloride and magnesium sulfate did not change during the COVID-19 pandemic. This result indicates that the prevalence of mild TPL with only oral ritodrine hydrochloride treatment decreased by lifestyle change due to the COVID-19 pandemic. As a result, the total number of patients with TPL during the pandemic decreased in our study. In contrast, the prevalence of severe TPL, which requires intravenous ritodrine hydrochloride, did not decrease during the pandemic. TPL and preterm birth are induced by various causes^7,11^, such as a previous history of TPL or preterm birth^12,13^, infection^7,14^, heavy labor^4–7^, and many other maternal complications. Our results indicate that a certain amount of mild TPL could be prevented by lifestyle change, including reduction of excessive physical activity.

The strength of our study was the large sample size compared to those in previous reports, as we evaluated the nationwide situation using real-world data in Japan. We also evaluated, in detail, the use of tocolytic agents as our dataset included medication records and the prescribed dosage of each medicine. A limitation of our study was our inability to estimate the extent by which people limited their physical or social activity. However, previous reports showed that people tend to be more sedentary and less active due to the COVID-19 pandemic worldwide^1^ and also in Japan^2^. Additionally, we could not obtain the data on gestational age at birth from our database. Since data on the diagnoses of some complications were obtained based on ICD-10 codes alone, the number of cases might be overestimated. However, we excluded suspected disease codes and combined the available information on applied medicine or procedure with ICD-10 codes to improve the diagnostic accuracy of the study.

## Conclusion

This study showed that the prevalence of TPL and preterm birth were significantly lower during the COVID-19 pandemic than in the pre-pandemic period. Neonatal outcomes were also improved during the pandemic than in the pre-pandemic period. Although the direct influence of a sedentary lifestyle on TPL remains controversial, our results suggest that maternal lifestyle changes due to the pandemic might have ameliorated TPL and consequently, reduced the risk of preterm birth.

## Data Availability

The data that support the findings of this study are available from the corresponding author (S.T.) upon reasonable request.
